# Striatiguttulaceae, a new pleosporalean family to accommodate *Longicorpus* and *Striatiguttula* gen. nov. from palms

**DOI:** 10.3897/mycokeys.49.30886

**Published:** 2019-04-01

**Authors:** Sheng-Nan Zhang, Kevin D. Hyde, E.B. Gareth Jones, Rajesh Jeewon, Ratchadawan Cheewangkoon, Jian-Kui Liu

**Affiliations:** 1 Center for Bioinformatics, School of Life Science and Technology, University of Electronic Science and Technology of China, Chengdu 611731, P.R. China; 2 Guizhou Key Laboratory of Agricultural Biotechnology, Guizhou Academy of Agricultural Science, Guiyang 550006, P.R. China; 3 Department of Entomology and Plant Pathology, Faculty of Agriculture, Chiang Mai University, Chiang Mai 50200, Thailand; 4 Center of Excellence in Fungal Research, Mae Fah Luang University, Chiang Rai 57100, Thailand; 5 Nantgaredig 33B St. Edwards Road, Southsea, Hants, UK; 6 Department of Health Sciences, Faculty of Science, University of Mauritius, Reduit, Mauritius, 80837, Mauritius

**Keywords:** 6 new taxa, divergence times, Dothideomycetes, epitype, sexual morphs

## Abstract

Palms represent the most morphological diverse monocotyledonous plants and support a vast array of fungi. Recent examinations of palmicolous fungi in Thailand led to the discovery of a group of morphologically similar and interesting taxa. A polyphasic approach based on morphology, multi-gene phylogenetic analyses and divergence time estimates supports the establishment of a novel pleosporalean family Striatiguttulaceae, which diversified approximately 39 (20–63) MYA (crown age) and 60 (35–91) MYA (stem age). Striatiguttulaceae is characterized by stromata or ascomata with a short to long neck, trabeculate pseudoparaphyses and fusiform to ellipsoidal, 1–3-septate ascospores, with longitudinal striations and paler end cells, surrounded by a mucilaginous sheath. Multi-gene phylogenetic analysis showed that taxa of Striatiguttulaceae form a well-supported and distinct monophyletic clade in Pleosporales, and related to *Ligninsphaeriaceae* and *Pseudoastrosphaeriellaceae*. However, these families can be morphologically demarcated by the slit-like ascomata and extremely large ascospores in *Ligninsphaeriaceae* and the rather narrow fusiform ascospores in *Pseudoastrosphaeriellaceae*. Eight strains of Striatiguttulaceae formed two monophyletic sub-clades, which can be recognized as *Longicorpus***gen. nov.** and *Striatiguttula***gen. nov.** Morphologically, the genus *Longicorpus* can be differentiated from *Striatiguttula* by its elongated immersed ascomata and fusiform ascospores with relatively larger middle cells and paler end cells. Two new species *Striatiguttulanypae* and *S.phoenicis*, and one new combination, *Longicorpusstriataspora* are introduced with morphological details, and phylogenetic relationships are discussed based on DNA sequence data.

## Introduction

Fungi associated with palms have been intensively investigated by Hyde and his co-workers (Goh and Hyde 1996, Fröhlich and Hyde 2000, Hyde and Alias 2000, Hyde et al. 2000, Yanna et al. 2001a,b,c, Taylor and Hyde 2003, Hyde et al. 2007), and provided a significant contribution to their diversity and taxonomy. There have been a number of interesting studies on palm fungi. For example, Fröhlich and Hyde (1999) reviewed the biodiversity of palm fungi in the tropics, and proposed the ratio of host specific fungi to palm species as 33 to 1 rather than the general ratio of 6 to 1 for all plants proposed by Hawksworth (1991). Taylor et al. (2000) investigated biogeographical distribution of microfungi from temperate and tropical palms, and found different fungal assemblages from these two regions, and also revealed that the difference was more related to climatic influences than hosts sampled. Subsequently, Yanna et al. (2001b, 2002) studied fungal communities and succession of palms, and pointed out that fungal species compositions were distinct on different hosts and at different sites, and even differed from different palm tissues. In addition, some studies were dedicated to endophytic palmicolous fungi (Rodrigues and Samuels 1990, Taylor et al. 1999, Fröhlich et al. 2000, Hyde and Soytong 2008, Pinruan et al. 2010a, Mahmoud et al. 2017) and pathogens (Fröhlich et al. 1997, Hyde and Cannon 1999, Elliott et al. 2010, Mohammadi 2014). Other studies have focused on fungi on peat swamp palms (Pinruan et al. 2002, 2007, 2008, 2010b, 2014, Pinnoi et al. 2003) and from mangrove palms (Suetrong et al. 2009, Loilong et al. 2012, Zhang et al. 2018). All these examples indicate that species are diverse and palms harbour numerous undescribed microfungi.

Ascomycetes from palms are a very diverse assemblage and the best represented family is *Xylariaceae* (Xylariales, Sordariomycetes), with three commonly recorded genera Anthostomella (Xylariaceae), Linocarpon (Linocarpaceae) and Oxydothis (Oxydothidaceae) (Taylor and Hyde 2003, Hidayat et al. 2006, Konta et al. 2016b, 2017). In recent years, a series of Dothideomycetes from palms were described as new on the basis of morphology and phylogenetic analyses, such as astrosphaeriella-like species (recognized as three groups: *Astrosphaeriellopsis*, *Astrosphaeriellaceae* and *Pseudoastrosphaeriellaceae*) and species of Botryosphaeria (Botryosphaeriaceae), Fissuroma (Aigialaceae), Neodeightonia (Botryosphaeriaceae) and Roussoella (Roussoellaceae) (Liu et al. 2010, 2011a,b, 2012, 2014, Phookamsak et al. 2015, Konta et al. 2016a,c, Wanasinghe et al. 2018). The diversity of palmicolous ascomycetes recovered can in part be due to the wide range of hosts and habitats sampled, the latter including terrestrial, freshwater, and marine or mangrove ecosystems. There are four palm species encountered as mangrove associates in Asia (Tomlinson 1986): *Calamuserinaceus* (Becc.) J.Dransf., *Nypafruticans* Wurmb., *Oncospermatigillarium* (Jack) Ridl. and *Phoenixpaludosa* Roxb. Loilong et al. (2012) documented the greatest biodiversity of fungi on *N.fruticans* listing 135 taxa (90 Ascomycota, three Basidiomycota and 42 asexual taxa), of which 97 taxa were described (Hyde 1992a,b, Hyde et al. 1999, Hyde and Alias 2000, Pilantanapak et al. 2005, Hyde and Sarma 2006) with support from DNA sequence data (Suetrong et al. 2015). Nevertheless, few studies have focused on fungi growing on *Phoenixpaludosa*, where *Lignincolaconchicola*, *Kirschsteiniotheliaphoenicis* and *Acuminatisporapalmarum* were recently reported (Liu et al. 2011a, Hyde et al. 2018, Zhang et al. 2018).

*Nypafruticans* is an ancient palm that grows in brackish water, while *Phoenixpaludosa* is found in the upper parts of mangroves and tolerates salt water, with both occurring in Thailand mangrove sites. In an ongoing study on the taxonomy of fungi occurring on palms, we collected fungi colonizing these two palm hosts from different mangrove sites in Thailand. Interestingly, a group of ascomycetes recovered appears to be new to science based on morphology and multi-gene phylogenetic evidence. The aim of this study was to characterize the novel taxa and investigate their phylogenetic relationships in the order Pleosporales, as well as apply the divergence times as additional evidence, especially in higher taxa ranking, for the establishment of new family Striatiguttulaceae.

## Materials and methods

### Specimen collection, examination and single spore isolation

Decayed rachides or petioles of *Nypafruticans* and *Phoenixpaludosa* were collected from Chanthaburi, Krabi and Ranong provinces in Thailand. The collected specimens were washed under running water and examined via laboratory procedures as outlined by Jones and Hyde (1988). Morphological characters were observed using a Carl Zeiss stereo microscope fitted with an AxioCam ERC 5S camera and photographed by a Nikon ECLIPSE 80i compound microscope fitted with a Canon EOS 600D digital camera. Free hand sections of fruiting bodies were made into slides within water mounts and observed under Motic SMZ 168 stereo microscope. Measurements were taken by Tarosoft Image Frame Work program v. 0.9.7 and images used for figures processed with Adobe Photoshop CS6 Extended v. 13.0 software. Isolations were obtained from single spores as described in Choi et al. (1999). New taxa were established based on recommendations outlined by Jeewon and Hyde (2016). The strains isolated in this study were deposited in Mae Fah Luang University Culture Collection (MFLUCC) and Guizhou Culture Collection (GZCC). Herbarium specimens were deposited at the herbaria of Mae Fah Luang University (MFLU), Chiang Rai, Thailand and Kunming Institute of Botany Academia Sinica (HKAS), Kunming, China. MycoBank numbers (Crous et al. 2004) and Facesoffungi numbers (Jayasiri et al. 2015) are provided.

### DNA extraction, PCR amplification and sequencing

Fungal genomic DNA was extracted from fresh mycelia scraped from the margin of a colony on PDA that was incubated at 25 °C–28 °C for 30 days, followed by the Ezup Column Fungi Genomic DNA Purification Kit (Sangon Biotech (Shanghai) Co., Ltd, China) following the manufacturer’s instructions. Two partial rDNA genes and two protein coding genes were used in this study: the large subunit of the nuclear ribosomal RNA genes (LSU), the small subunit of the nuclear ribosomal RNA (SSU), the translation elongation factor 1-alpha (*TEF1α*) and the second largest subunit of RNA polymerase II (*RPB2*). The primers used were LR0R and LR5 for LSU (Vilgalys and Hester 1990), NS1/NS4 for SSU (White et al. 1990), EF1-983F/EF1-2218R for *TEF1α* (Rehner and Buckley 2005) and fRPB2-5F/fRPB2-7cR for *RPB2* (Liu et al. 1999). The amplification reactions were performed in 25μL of PCR mixtures containing 9.5μL ddH_2_O, 12.5μL 2× PCR MasterMix (TIANGEN Co., China), 1μL DNA temple and 1μL of each primer. The PCR thermal cycle program for LSU, SSU and *TEF1α* amplification were as follows: initial denaturing step of 94 °C for 3 min, followed by 40 cycles of denaturation at 94 °C for 45 seconds, annealing at 56 °C for 50 seconds, elongation at 72 °C for 1 min, and final extension at 72 °C for 10 min. The PCR thermal cycle program for the partial RNA polymerase second largest subunit (*RPB2*) was followed as initially 95 °C for 5 min, followed by 40 cycles of denaturation at 95 °C for 1 min, annealing at 52 °C for 2 min, elongation at 72 °C for 90 seconds, and final extension at 72 °C for 10 min. Purification and sequencing of PCR products were carried out with primers mentioned above at Sangon Biotech (Shanghai) Co., Ltd, China.

### Sequence alignment and phylogeny analyses

A concatenated data set of LSU, SSU, *TEF1α* and *RPB2* sequences was used for phylogenetic analyses with the inclusion of reference taxa from GenBank (Table 1). Sequences were aligned using MAFFT v.7 (http://mafft.cbrc.jp/alignment/server/) (Katoh and Standley 2013) and then checked visually and manually optimized using BioEdit v.7.0.9 (Hall 1999). Representative families in Pleosporales and several major groups in Dothideomycetes were included in our analyses, and taxa in Arthoniomycetes were selected as outgroup. A maximum likelihood (ML) analysis was performed at the CIPRES web portal (Miller et al. 2010) using RAxML v.7.2.8 as part of the “RAxML-HPC Blackbox (8.2.10)” tool (Stamatakis 2006, Stamatakis et al. 2008). A general time-reversible model (GTR) was applied with a discrete GAMMA distribution and four rate classes. Fifty thorough ML tree searches were carried out in RAxML v.7.2.7 under the same model. One thousand non-parametric bootstrap iterations were run with the GTR model and a discrete gamma distribution. The resulting replicates were plotted on to the best scoring tree obtained previously.

**Table 1.** Taxa used in this study and their GenBank accession numbers. The type species of each genus are marked with superscript ^T^ and ex-type strains are in bold.

Taxa	Strain / Culture	GenBank Accession numbers			
		LSU	SSU	*TEF1α*	*RPB2*
* Acrocordiopsis patilii *	BCC28167	GU479773	GU479737	–	GU479812
* Acrocordiopsis patilii * ^T^	BCC28166	GU479772	GU479736	–	GU479811
* Acuminatispora palmarum *	MFLUCC 18-0460	MH390438	MH390402	MH399249	MH399252
*** Acuminatispora palmarum *** ^T^	MFLUCC 18-0264	MH390437	MH390401	MH399248	–
* Aigialus grandis * ^T^	BCC18419	GU479774	GU479738	GU479838	GU479813
* Aigialus mangrovei *	BCC33563	GU479776	GU479741	GU479840	GU479815
* Aigialus parvus *	BCC 18403	GU479778	GU479744	GU479842	GU479817
* Aigialus rhizophorae *	BCC 33572	GU479780	GU479745	GU479844	GU479819
* Alternaria alternata *	CBS 916.96	DQ678082	DQ678031	DQ677927	DQ677980
*** Amniculicola lignicola *** ^T^	Ying01	EF493861	EF493863	–	EF493862
* Anteaglonium abbreviatum * ^T^	ANM 925a	GQ221877	–	GQ221924	–
*** Anteaglonium globosum ***	ANM 925.2	GQ221879	–	GQ221925	–
* Antealophiotrema brunneosporum * ^T^	CBS 123095	LC194340	–	LC194382	LC194419
* Aquasubmersa japonica *	KT 2862	LC061587	LC061582	–	LC194421
*** Aquasubmersa mircensis *** ^T^	MFLUCC 11-0401	JX276955	JX276956	–	–
* Arthonia dispersa *	UPSC2583	AY571381	AY571379	–	–
* Ascocratera manglicola * ^T^	BCC 09270	GU479782	GU479747	GU479846	GU479821
* Astrosphaeriella fusispora * ^T^	MFLUCC 10-0555	KT955462	–	–	KT955413
* Astrosphaeriella neofusispora *	MFLUCC 11-0161	KT955463	KT955444	–	KT955418
* Astrosphaeriella stellata *	KT998	AB524592	AB524451	–	–
* Astrosphaeriellopsis bakeriana *	MFLUCC 11-0027	JN846730	–	–	–
* Astrosphaeriellopsis bakeriana * ^T^	CBS 115556	GU301801	–	GU349015	–
*** Bimuria novae-zelandiae *** ^T^	CBS 107.79	AY016356	AY016338	DQ471087	DQ470917
* Botryosphaeria dothidea *	CMW 8000	KF766319	KF766233	–	–
* Byssothecium circinans * ^T^	CBS 675.92	AY016357	–	GU349061	DQ767646
* Capnodium coffeae *	CBS 147.52	DQ247800	DQ247808	DQ471089	DQ247788
* Caryospora minima *	–	EU196550	EU196551	–	–
*** Caryospora aquatica ***	MFLUCC 11-0008	MH057847	MH057850	–	–
* Cladosporium herbarum *	CBS 399.80	DQ678074	DQ678022	DQ677918	DQ677971
* Cryptocoryneum condensatum *	CBS 122629	LC194351	LC194309	LC096139	LC194433
*** Cryptocoryneum pseudorilstonei ***	CBS 113641	LC194364	LC194322	LC096152	LC194446
* Delitschia chaetomioides *	SMH 3253.2	GU390656	–	–	–
*** Delitschia didyma ***	UME 31411	DQ384090	AF242264	–	–
* Delitschia winteri *	CBS 225.62	DQ678077	–	–	DQ677975
* Dendrographa decolorans *	Ertz 5003 (BR)	NG_027622	AY548809	–	–
* Didymella exigua * ^T^	CBS 183.55	EU754155	EU754056	–	–
* Didymosphaeria rubi-ulmifolii *	MFLUCC 14-0023	KJ436586	KJ436588	–	–
* Dissoconium aciculare *	CBS 204.89	GU214419	GU214523	–	–
* Dothidotthia aspera *	CPC 12933	EU673276	EU673228	–	–
* Dothidotthia symphoricarpi * ^T^	CPC 12929	EU673273	EU673224	–	–
* Extremus antarcticus *	CCFEE 5312	KF310020	–	–	KF310086
* Fissuroma bambusae *	MFLUCC 11-0160	KT955468	KT955448	KT955430	KT955417
* Halotthia posidoniae * ^T^	BBH 22481	GU479786	–	–	–
*** Hermatomyces iriomotensis ***	MAFF 245730	LC194367	–	LC194394	LC194449
* Hypsostroma caimitalense *	GKM 1165	GU385180	–	–	–
* Hypsostroma saxicola * ^T^	SMH 5005	GU385181	–	–	–
* Hysterium angustatum *	CBS 236.34	FJ161180	GU397359	FJ161096	–
* Hysterobrevium smilacis *	CBS 114601	FJ161174	FJ161135	FJ161091	–
* Latorua caligans * ^T^	CBS 576.65	KR873266	–	–	–
* Latorua grootfonteinensis *	CBS 369.72	KR873267	–	–	–
* Lecanactis abietina *	Ertz 5068 (BR)	AY548812	AY548805	–	–
*** Longicorpus striataspora *** ^T^	MFLUCC 18-0267	MK035988	MK035973	MK034428	MK034436
* Longicorpus striataspora *	MFLUCC 18-0268	MK035989	MK035974	MK034429	MK034437
* Longicorpus striataspora *	MFLUCC 17-2515	MK035990	MK035975	MK034430	MK034438
* Longicorpus striataspora *	MFLUCC 17-2516	MK035991	MK035976	MK034431	MK034439
* Lepidosphaeria nicotiae *	CBS 101341	DQ678067	–	–	DQ677963
* Leptosphaeria doliolum * ^T^	CBS 505.75	GU301827	GU296159	GU349069	–
* Leptoxyphium fumago *	CBS 123.26	GU301831	GU214535	GU349051	GU371741
* Ligninsphaeria jonesii *	GZCC 15-0080	KU221038	–	–	–
*** Ligninsphaeria jonesii *** ^T^	MFLUCC 15-0641	KU221037	–	–	–
* Lindgomyces cinctosporae *	R56-1	AB522431	AB522430	–	–
* Lindgomyces ingoldianus * ^T^	ATCC 200398	AB521736	AB521719	–	–
* Lindgomyces rotundatus *	KT1096	AB521740	AB521723	–	–
* Lophiostoma macrostomoides *	GKM1033	GU385190	–	–	–
‘*Lophiotrema’ boreale*	CBS 114422	LC194375	–	LC194402	LC194457
* Lophiotrema lignicola *	CBS 122364	GU301836	GU296166	GU349072	–
* Lophiotrema nucula * ^T^	CBS 627.86	GU301837	GU296167	GU349073	GU371792
* Macrodiplodiopsis desmazieri * ^T^	CPC 24971	KR873272	–	–	–
* Massaria anomia *	CBS 591.78	GU301839	GU296169	–	GU371769
* Massaria gigantispora *	M26	HQ599397	HQ599447	HQ599337	–
* Massaria inquinans * ^T^	M19	HQ599402	HQ599444	HQ599342	HQ599460
* Massarina eburnea * ^T^	CBS 473.64	GU301840	GU296170	GU349040	GU371732
* Mauritiana rhizophorae * ^T^	BCC 28866	GU371824	–	GU371817	GU371796
* Melanomma pulvis-pyrius * ^T^	CBS 124080	GU456323	GU456302	GU456265	GU456350
* Murispora rubicunda * ^T^	IFRD 2017	FJ795507	GU456308	–	–
* Mycosphaerella graminicola *	CBS 292.38	DQ678084	DQ678033	–	DQ677982
*** Neoastrosphaeriella krabiensis *** ^T^	MFLUCC 11-0025	JN846729	JN846739	–	–
* Neodeightonia palmicola *	MFLUCC10-0822	HQ199222	HQ199223	–	–
* Neotestudina rosatii *	CBS 690.82	DQ384107	DQ384069	–	–
*** Nigrograna mackinnonii *** ^T^	CBS 674.75	GQ387613	–	–	KF015703
* Nigrograna marina *	CY 1228	GQ925848	–	–	GU479823
* Phaeosphaeria oryzae * ^T^	CBS 110110	GQ387591	GQ387530	–	KF252193
* Phoma herbarum * ^T^	CBS 276.37	DQ678066	DQ678014	DQ677909	DQ677962
Piedraia hortae var. hortae	CBS 480.64	GU214466	AY016349	–	DQ677990
* Pleomassaria siparia * ^T^	CBS 279.74	DQ678078	DQ678027	–	DQ677976
*** Pleospora herbarum *** ^T^	CBS 191.86	DQ247804	DQ247812	DQ471090	DQ247794
*** Polyplosphaeria fusca *** ^T^	KT 1616	AB524604	AB524463	–	–
* Preussia funiculata * ^T^	CBS 659.74	GU301864	–	–	–
* Prosthemium orientale *	KT1669	AB553748	AB553641	–	–
*** Pseudoastrosphaeriella africana ***	MFLUCC 11-0176	KT955474	KT955454	KT955436	KT955421
*** Pseudoastrosphaeriella bambusae ***	MFLUCC 11-0205	KT955475	–	KT955437	KT955414
*** Pseudoastrosphaeriella longicolla ***	MFLUCC 11-0171	KT955476	–	KT955438	KT955420
*** Pseudoastrosphaeriella thailandensis *** ^T^	MFLUCC 11-0144	KT955478	KT955457	KT955440	KT955416
*** Pseudotetraploa curviappendiculata *** ^T^	HC 4930	AB524608	AB524467	–	–
*** Quadricrura septentrionalis *** ^T^	HC 4984	AB524616	AB524475	–	–
* Racodium rupestre *	L346	EU048583	EU048575	–	–
* Roccella fuciformis *	Tehler 8171	FJ638979	–	–	–
*** Roussoella nitidula *** ^T^	MFLUCC 11-0182	KJ474843	–	KJ474852	KJ474859
* Roussoellopsis macrospora *	MFLUCC 12-0005	KJ474847	–	KJ474855	KJ474862
* Salsuginea ramicola *	KT2597.2	GU479801	GU479768	GU479862	GU479834
* Salsuginea ramicola * ^T^	KT 2597.1	GU479800	GU479767	GU479861	GU479833
*** Striatiguttula nypae *** ^T^	MFLUCC 18-0265	MK035992	MK035977	MK034432	MK034440
* Striatiguttula nypae *	MFLUCC 17-2517	MK035993	MK035978	MK034433	MK034441
* Striatiguttula nypae *	MFLUCC 17-2518	MK035994	MK035979	MK034434	–
*** Striatiguttula phoenicis *** ^T^	MFLUCC 18-0266	MK035995	MK035980	MK034435	MK034442
*** Tetraplosphaeria sasicola *** ^T^	KT563	AB524631	AB524490	–	
*** Trematosphaeria pertusa *** ^T^	CBS 122371	FJ201992	–	–	GU371801
*** Triplosphaeria maxima *** ^T^	KT 870	AB524637	AB524496	–	–
* Ulospora bilgramii * ^T^	CBS 101364	DQ678076	DQ678025	DQ677921	DQ677974
* Verruculina enalia * ^T^	BCC 18401	GU479802	–	GU479863	GU479835
* Wicklowia aquatica *	AF289-1	GU045446	–	–	–
*** Wicklowia aquatica *** ^T^	F76-2	GU045445	GU266232	–	–
* Zopfia rhizophila * ^T^	CBS 207.26	DQ384104	–	–	–

Abbreviations: **ATCC**: American Type Culture Collection, Virginia, USA; **BBH**: Biotec Bangkok Herbarium, Thailand; **BCC**: BIOTEC Culture Collection, Bangkok, Thailand; **CBS**: Centraal bureau voor Schimmel cultures, Utrecht, The Netherlands; **CPC**: Collection of Pedro Crous housed at CBS; **DAOM**: Plant Research Institute, Department of Agriculture (Mycology), Ottawa, Canada; **GZCC**: Guizhou Culture Collection; **IFRDCC**: Culture Collection, International Fungal Research & Development Centre, Chinese Academy of Forestry, Kunming, China; **JCM**: the Japan Collection of Microorganisms, Japan; **MAFF**: Ministry of Agriculture, Forestry and Fisheries, Japan; **MFLU**: Mae Fah Luang University Herbarium Collection; **MFLUCC**: Mae Fah Luang University Culture Collection, Chiang Rai, Thailand. **ANM**: A.N. Miller; **GKM**: G.K. Mugambi; **JK**: J. Kohlmeyer; **KT**: K. Tanaka; **SMH**: S.M. Huhndorf.

Maximum parsimony (MP) analyses were performed using the heuristic search option with 1000 random taxa additions and tree bisection and reconnection (TBR) as the branch-swapping algorithm. All characters were unordered and of equally weight; gaps were treated as missing data. Maxtrees setting was 1000, and zero-length branches were collapsed, and all parsimonious trees were saved. Clade stability was assessed using a bootstrap (BT) analysis with 1000 replicates, each with 10 replicates of random stepwise addition of taxa (Hillis and Bull 1993). Tree length [TL], Consistency index [CI], Retention index [RI], Rescaled consistency index [RC], Homoplasy index [HI] were calculated.

The Bayesian analysis was performed using PAUP v.4.0b10 (Swofford 2002) and MrBayes v.3.1.2 (Ronquist and Huelsenbeck 2003). The best model for different genes partition in the concatenated data set was determined by MrModeltest 2.3 (Nylander 2004). Posterior probabilities (Rannala and Yang 1996) were determined by Markov Chain Monte Carlo sampling (MCMC) (Larget and Simon 1999) in MrBayes v.3.1.2. Four simultaneous Markov chains were run for 10 million generations and trees were sampled every 1000^th^ generation, thus 10,000 trees were obtained. The suitable burn-in phases were determined by inspecting likelihoods and parameters in Tracer version 1.6 (Rambaut et al. 2013). Based on the tracer analysis, the first 1,000 trees representing 10% were discarded as the burn-in phase in the analysis. The remaining trees were used to calculate posterior probabilities in the majority rule consensus tree (critical value for the topological convergence diagnostic set to 0.01). Phylogenetic tree was visualized by FigTree v.1.4.2 (Rambaut 2014), and the alignment is deposited in TreeBASE under the accession number TB2: S23392 (http://purl.org/phylo/treebase/phylows/study/TB2:S23392).

### Divergence times estimates

One secondary data and two fungal fossil calibrations were used in this study. The split between Arthoniomycetes and Dothideomycetes was selected as a secondary calibration point referring to previous evolutionary molecular studies (Gueidan et al. 2011, Prieto and Wedin 2013, Beimforde et al. 2014, Pérez-Ortega et al. 2016, Phukhamsakda et al. 2016), with a mean of 300 MYA and standard deviation (SD) of 50 MYA in a normal posterior distribution. Simultaneously, one ascomycete fossil *Metacapnodiaceae* (Schmidt et al. 2014), was used as the common ancestor of Capnodiales, with constraint of mean 100 MYA and SD 150 MYA in a normal posterior distribution (Pérez-Ortega et al. 2016, Hongsanan et al. 2016, Phukhamsakda et al. 2016, Liu et al. 2017). Whereas the fossil *Margaretbarromycesdictyosporus* (Mindell et al. 2007, Berbee and Taylor 2010, Taylor et al. 2015) was used to calibrate the Aigialus (Aigialaceae) crown, with an offset of 35 MYA in a gamma distribution (Phukhamsakda et al. 2016). Divergence time estimates were carried out by BEAST v 1.8.0 (Drummond et al. 2012). Aligned sequence data were partitioned separately for LSU, SSU, *TEF1α* and *RPB2* data set, and loaded to prepare an XML file constructed with BEAUti v1.8.0. The substitution models, clock models and the tree prior parameters were set to be linked. The nucleotide substitution model was set to GTR (Generalized Time Reversible) + Gamma + Invariant sites, with estimated base frequencies, four gamma categories and without partitions. An uncorrelated relaxed clock model (Drummond et al. 2007) with a lognormal distribution of rates for each gene estimate was used for the analyses. We used a Yule tree prior, which assumes a constant speciation rate per lineage, and a randomly generated starting tree. The analysis was run for 100 million generations and parameters were sampled every 10,000 generations. Tracer v.1.6 (Rambaut et al. 2013) was used to analyze the trace files, and the acceptable effective sample sizes (ESS) values were greater than 200. Maximum clade creditability (MCC) trees were annotated using TreeAnnotator v1.8.0 and then visualized in FigTree v.1.4.2 (Rambaut 2014).

## Results

### Phylogenetic results

**Figure 1.**
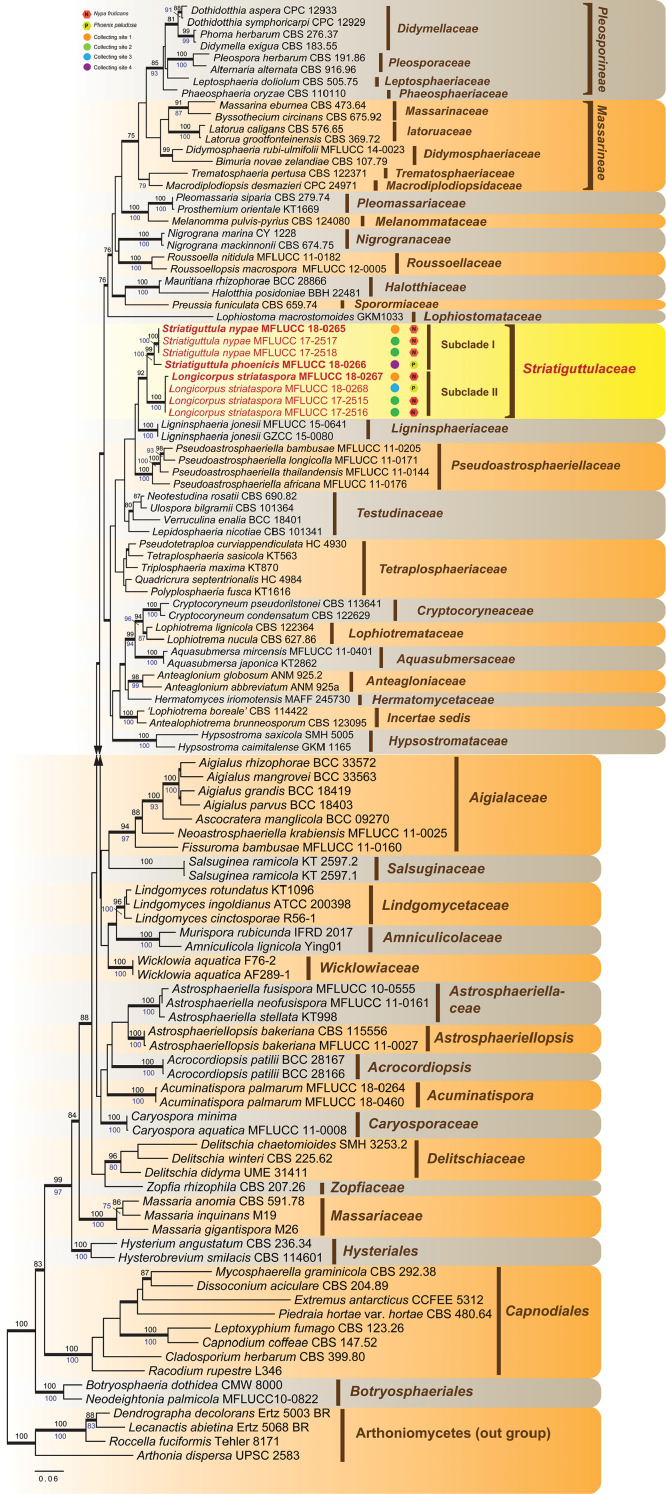
RAxML tree of Pleosporales based on analysis of combined LSU, SSU, *TEF1α* and *RPB2* sequence data. Bootstrap values for ML and MP equal to or greater than 75% are placed above and below the branches respectively. Branches with Bayesian posterior probabilities (PP) from MCMC analysis equal or greater than 0.95 are in bold. Newly generated sequences are indicated in red.

The multi-gene dataset comprised 113 taxa and 4113 characters after alignment (LSU: 919 bp; SSU: 1245 bp; *TEF1α*: 929 bp; *RPB2*: 1020 bp) including gaps. RAxML, MP and Bayesian analyses were conducted and resulted in generally congruent topologies, and the familial assignments are similar to previous work (Hashimoto et al. 2017, Liu et al. 2017). Maximum parsimony analyses indicated that 2,302 characters were constant, 355 variable characters parsimony uninformative and 1,456 characters are parsimony-informative. A heuristic search yield four equally most parsimonious trees (TL = 10905, CI = 0.278, RI = 0.561, RC = 0.156, HI = 0.722). The combined dataset provided higher confidence values for the familial level than those of the individual gene trees (data not shown), and RAxML analysis based on LSU, SSU, *TEF1α* and *RPB2* yielded a best sorting tree (Figure 1) with a final optimization likelihood value of -52455.532059.

The eight newly generated strains clustered together and positioned outside the two suborders (Massarineae and Pleosporineae) of Pleosporales, and formed a well-supported monophyletic clade and represented as a new linage of Pleosporales. The phylogeny also revealed that this clade is close to *Ligninsphaeriaceae*, *Pseudoastrosphaeriellaceae*, *Testudinaceae* and *Tetraplosphaeriaceae*, and can be recognized as a novel family (Striatiguttulaceae). Furthermore, the eight strains formed two well-supported monophyletic sub-clades, which can be identified as two new genera (*Longicorpus* and *Striatiguttula*) with three species (*Longicorpusstriataspora*, *Striatiguttulanypae* and *S.phoenicis*).

### Divergence time estimates

**Figure 2.**
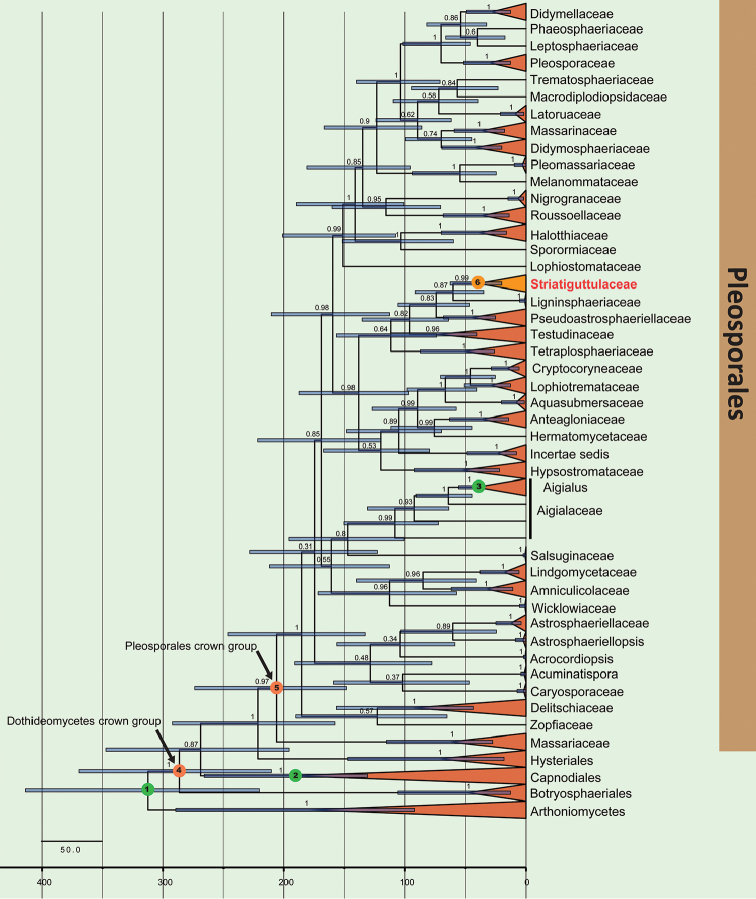
Maximum clade credibility (MCC) tree with divergence times estimates for Pleosporales and selected groups in Dothideomycetes, obtained from a Bayesian approach (BEAST) using one secondary and two fossil calibrations. Numbers at nodes indicate posterior probabilities (pp) for node support; bars correspond to the 95% highest posterior density (HPD) intervals. Numbers inside green circles indicate nodes used for calibrations: 1) the split of Arthoniomycetes and Dothideomycetes; 2) *Metacapnodiaceae*; 3) *Margaretbarromycesdictyosporus*.

The maximum clade credibility (MCC) tree with divergence estimates (Figure 2) obtained through BEAST was topologically identical to those recovered by Bayesian and ML procedures with regards to the placement Pleosporales and several major lineages within Dothideomycetes. The mean dates of Pleosporales crown corroborate reported estimates (Phukhamsakda et al. 2016, Liu et al. 2017, 2018) are provided in Table 2. The results showed that the new family Striatiguttulaceae diverged approximately 60 (35–91) MYA, which is line with recommendations for ranking families proposed in related studies (Hyde et al. 2017, Liu et al. 2017).

**Table 2.** Divergence time estimates of Pleosporales and selected lineages of Dothideomycetes obtained from a Bayesian approach (BEAST) on basis of three calibrations. For each divergence, the median and the 95% highest posterior density (HPD) are provided. Divergence times are provided in millions of years (MYA).

**Nodes**	**Crown group**	**Divergence times**				
		**This study**		**Phukhamsakda et al. (2016)**	**Liu et al. (2017)**	**Liu et al. (2018)**
		**Crown age**	**Stem age**	**Crown age**		
1	Arthoniomycetes-Dothideomycetes	312 (220–413)	–	317	–	310~320
2	Capnodiales	195 (131–266)	269 (196–347)	147	216/ (151–283)	~120
3	* Aigialus *	41 (35–56)	64 (44–91)	39	–	~50
4	Dothideomycetes	286 (210–369)	312 (220–413)	293 ~(210–370)	341 (257–425)	255 (166–344)
5	Pleosporales	206 (148–274)	221 (158–292)	211 ~(140–270)	204 (148–260)	195 (124–271)
6	Striatiguttulaceae	39 20–63)	60 (35–91)	–	–	–

### Taxonomy

#### Striatiguttulaceae

Taxon classificationFungiPleosporalesStriatiguttulaceae

S.N.Zhang, K.D.Hyde & J.K.Liu, fam. nov.

MB828272

##### Etymology.

Name refers to the name of the type genus.

##### Description.

*Saprobic* on palms distributed in mangrove habitats. **Sexual morph**: *Stromata* black, scattered to gregarious, immersed beneath host epidermis, and erumpent to superficial, with a papilla or a short to long neck, ampulliform, subglobose or conical, uni-loculate or bi-loculate, coriaceous to carbonaceous, ostiolate, periphysate, papillate, clypeate or not clear, glabrous or somewhat interwoven pale brown hyphae or setae. *Peridium* composed of several brown to hyaline cell layers. *Hamathecium* of trabeculate pseudoparaphyses. *Asci* 8-spored, bitunicate, cylindric-clavate, pedicellate. *Ascospores* hyaline to brown, uniseriate to biseriate or triseriate, fusiform or ellipsoidal, 1–3-septate, striate, guttulate, with paler end cells and surrounded by a mucilaginous sheath. **Asexual morph**: Undetermined.

##### Type genus.

*Striatiguttula* S.N.Zhang, K.D.Hyde & J.K.Liu.

##### Notes.

The family Striatiguttulaceae is introduced to accommodate two new genera *Longicorpus* and *Striatiguttula*, characterized by the immersed, and erumpent to superficial stromata, with a papilla or a short to long neck, trabeculate pseudoparaphyses, bitunicate asci, and hyaline to brown, fusiform to ellipsoidal, striate, guttulate, 1–3-septate ascospores, with paler end cells and surrounded by a mucilaginous sheath. Members of Striatiguttulaceae are morphologically similar to the genera *Leptosphaeria* and *Trematosphaeria*, but they are phylogenetically distinct and also differ in ascospores characteristics and the latter two have coriaceous, heavily pigmented thick-walled peridium. Multi-gene phylogenetic analyses revealed a close relationship of Striatiguttulaceae to *Ligninsphaeriaceae* and *Pseudoastrosphaeriellaceae*. However, Striatiguttulaceae differs from *Pseudoastrosphaeriellaceae* as the latter has 1–3-septate or 2–5-septate ascospores, which are narrowly fusiform with acute ends and all cells are concolorous. The slit-like ascomata and broad fusiform, 1-septate, rather large ascospores (79–121 × 14–23 µm) in *Ligninsphaeriaceae* (Zhang et al. 2016) are distinct from those found in Striatiguttulaceae. Additionally, a divergence time estimate analysis indicated that the crown age 39 (20–63) MYA and stem age 60 (35–91) MYA of Striatiguttulaceae, match with the recommendations of using divergence times to recognize families in Liu et al. (2017). Attempts were made to culture the asexual morph in order to build comprehensive familial concept for Striatiguttulaceae, but it was not successful. Further morphological investigations together with more molecular data are needed.

#### Striatiguttula

Taxon classificationFungiPleosporalesStriatiguttulaceae

S.N.Zhang, K.D.Hyde & J.K.Liu, gen. nov.

MB828273

##### Etymology.

Name refers to the striate and guttulate ascospores.

##### Description.

*Saprobic* on palms which are distributed in mangrove habitats. **Sexual morph**: *Stromata* black, scattered to gregarious, immersed beneath host epidermis, and erumpent to superficial, with a papilla or a short to long neck, ampulliform, subglobose or conical, uni-loculate or bi-loculate, coriaceous to carbonaceous, ostiolate, periphysate, papillate, clypeate or not, glabrous or somewhat interwoven pale brown hyphae or setae, lying at apex of the neck. *Peridium* thin, composed of several pale brown to hyaline angular cells. Wall of the neck having elongated angular cells. *Hamathecium* filament thin, trabeculate pseudoparaphyses, septate, branched, anastomosing, embedded in a gelatinous matrix. *Asci* 8-spored, bitunicate, cylindric-clavate, pedicellate, apically rounded, with an ocular chamber. *Ascospores* hyaline to brown, uniseriate to biseriate or triseriate, fusiform to ellipsoidal, 1–3-septate, constrict, the middle cells slightly swollen towards the central septa, striate, guttulate, end cells slightly paler or not, surrounded by a mucilaginous sheath. **Asexual morph**: Undetermined.

##### Type species.

*Striatiguttulanypae* S.N.Zhang, K.D.Hyde & J.K.Liu.

#### Striatiguttula nypae

Taxon classificationFungiPleosporalesStriatiguttulaceae

S.N.Zhang, K.D.Hyde & J.K.Liu, sp. nov.

MycoBank number: MB828274

Figure 3

##### Etymology.

The epithet reflects the genus name of the host plant *Nypafruticans*, from which the specimens were collected.

##### Type.

THAILAND. Ranong: Ranong, on decayed rachis of *Nypafruticans* Wurmb (*Arecaceae*), 3 December 2016, S.N.Zhang, SNT44 (holotype: MFLU 18–1576; isotype: HKAS 97480; ex-type living culture MFLUCC 18–0265 = GZCC 18–0005).

##### Description.

*Saprobic* on mangrove palm *Nypafruticans*. **Sexual morph**: *Stromata* in vertical section 240–380 µm high, 195–385 µm diameter, (x̄ = 318.2 × 289.0 µm, n = 15), black, scattered, gregarious, immersed beneath host epidermis, and erumpent to superficial, with a papilla or short to long neck up to 550 µm, subglobose or conical, uni-loculate or bi-loculate, coriaceous to carbonaceous, ostiolate, periphysate, papillate and clypeate, glabrous or somewhat interwoven pale brown hyphae or with setae, lying at apex of the neck. *Peridium* 9–16 µm thin, composed of several pale brown to hyaline angular cells, compressed and pallid inwardly. Wall of the clypeus composed of brown cells of *textura* epidermoidea and dark brown host tissue. Wall of the neck with thicker and elongated angular cells. *Hamathecium* 1–2 µm wide, trabeculate pseudoparaphyses, septate, branched, filamentous, anastomosing, embedded in a gelatinous matrix. *Asci* 64–145 × 8–17 µm, (x̄= 106.3 × 13.8 µm, n = 30), 8-spored, bitunicate, fissitunicate, cylindric-clavate, pedicellate, apically rounded, with an ocular chamber. *Ascospores* 18–26 × 4–6 µm, (x̄ = 22.2 × 5.3 µm, n = 50), hyaline to brown, uniseriate to biseriate or triseriate, fusiform, 1–3-septate, constricted at the central septum, the upper middle cell slightly swollen towards the central septum, straight or slightly curved, striate, guttulate, end cells slightly paler, surrounded by a mucilaginous sheath. **Asexual morph**: Undetermined.

**Figure 3.**
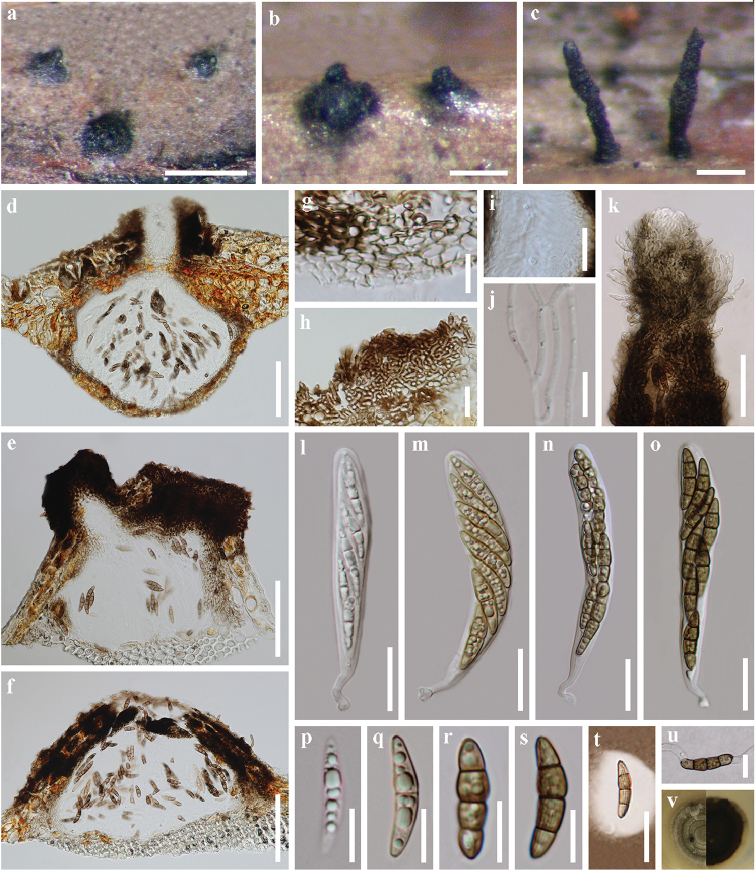
*Striatiguttulanypae* (holotype MFLU 18–1576, paratype MFLU 18–1578). **a–c** Appearance of stromata on host surface **d–f** vertical section through a stroma **g** structure of peridium **h** structure of clypeus near the ostiole, composed of epidermoidea cells and host tissue **i** ostiole with periphyses **j** pseudoparaphyses **k** apex of the neck, with somewhat interwoven pale brown hyphae or setae **l–o** ascus **p–s** ascospores **t** ascospore in India ink and presenting a clear mucilaginous sheath **u** germinating ascospore **v** colony on PDA. Scale bars: 500 μm (**a**), 200 μm (**b, c**), 100 μm (**d–f**), 10 μm (**g, p–s, u**), 20 μm (**h, i, l–o, t**), 50 μm (**k**).

##### Culture characteristics.

Colonies on PDA attaining 15 mm diam. within 21 days at 25 °C under natural light, velvety, centrally raised, greenish grey or greyish olivaceous, reverse dull green or grey olivaceous, with a margin of translucent, milky white to hyaline mycelia.

##### Additional specimens examined.

Thailand. Krabi: near Pali, Mueang Krabi District, on submerged decaying rachis of *Nypafruticans* Wurmb (*Arecaceae*), 30 August 2017, S.N.Zhang, SNT207 (paratype: MFLU 18–1577; living culture MFLUCC 17–2517 = GZCC 18–0006); Thailand. Krabi: near Pali, Mueang Krabi District, on submerged decaying rachis of *Nypafruticans* Wurmb (*Arecaceae*), 30 August 2017, S.N.Zhang, SNT208 (paratype: MFLU 18–1578; living culture MFLUCC 17–2518 = GZCC 18–0007).

##### Habitat and distribution.

Inhabiting Thai mangrove forests, Andaman sea (west) coastline, Thailand.

##### Notes.

*Striatiguttulanypae* varies in ascomatal appearance, mostly immersed beneath the plant surface, sometimes visible as a papilla or dome-shaped area on the plant surface, and becomes erumpent to superficial, with a papilla or a short to long neck. The typical morphological characters of *S.nypae* are the appearance of stromata, with interwoven pale brown hyphae or setae at the apex of the neck, and the hyaline to brown, 1–3-septate, fusiform ascospores, striate, guttulate, with slightly paler end cells and a mucilaginous sheath. We have compared *Striatiguttulanypae* to previously encountered species on *Nypafruticans*, and several morphologically similar mangrove fungal species. However, the striation of ascospores can be a reliable morphological character to distinguish *Striatiguttulanypae* from *Astrosphaeriellanipicola* (Hyde and Fröhlich 1998), *A.nypae* (Hyde 1992a) and *Leptosphaeria* spp. (Spegazzini 1881, Cribb and Cribb 1955, Hyde et al. 1999, Pang et al. 2011), which are characterized by one or three septa and hyaline or brown ascospores. The presence of erumpent to superficial stromata, the number of septa and size of ascospores in *S.nypae* are also different from *Trematosphaeria* spp. (Table 3), despite being quite similar in ascospore morphology. In addition, the phylogenetic analysis showed that the three isolates of *Striatiguttulanypae* clustered together and were distinct from *S.phoenicis*.

**Table 3.** Morphological comparison of three new species to *Trematosphaerialineolatispora*, *T.mangrovis* and *T.striataspora*.

Taxa	Ascomata		Peridium (µm)	Pseudoparaphyses (µm)	Asci (µm)	Ascospores		References
	Ascomata morphology	(high × diam. μm)				Ascospores morphology	Ascospores size (µm)	
* Longicorpus striataspora *	Immersed, erumpent, ampulliform, subglobose or conical, CA	300–500 × 230–560	11–15	1.5	85–160 × 10–17	Fusiform, 1–3-septate, CC	24–45 × 7–8.8	This study
* Striatiguttula nypae *	Immersed and erumpent to superficial, subglobose or conical, uni-loculate or bi-loculate, CA	240–380 × 195–385	9–16	1–2	64–145 × 8–17	Fusiform, 1–3-septate, CC	18–26 × 4–6	This study
* Striatiguttula phoenicis *	Immersed, erumpent, ampulliform, subglobose, uni-loculate, CB	195–580 × 135–390	10–24	1–2	89–141 × 12–18	Fusiform to ellipsoidal, 1–3-septate, CC but nearly concolorous	20–29 × 6–10	This study
*Trematosphaerialineolatispora* K.D. Hyde	Immersed with a flattened base, conical to subglobose, clypeate, ostiolate, papillate	90–180 × 216–360	up to 25	2–4	120–204 × 14–18	Fusiform, mostly 5-septate; CC	34–48 × 7–10	Hyde 1992b
*Trematosphaeriamangrovis* Kohlm.	Semi-immersed, conical or subglobose, papillate	380–750 × 450–800	64–88	1.6–2.2	190–220 × 20–22	Broad fusiform or ellipsoidal, 3-septate, CC but no striations	30–35.6–41 × 10–11.8–13 (–16.5)	Kohlmeyer 1968
*Trematosphaeriastriataspora* K.D. Hyde	Developing amongst the host cortical cells beneath the host epidermis, ampulliform, subglobose or conical, CA	176–355 × 352–528	42–57 (clypeus), thin-walled	0.8–2.1	99–173 × 11–23	Fusiform, 3(–6)- septate, CC	31–38 × 6–9	Hyde 1988

**CA**: (Characteristics A) clypeate, ostiolate, periphysate, papillate; **CB**: (Characteristics B) ostiolate, periphysate, papillate; **CC**: (Characteristics C) central cells larger, brown, end cells smaller and paler, ascospore wall covered in distinct longitudinal striations, and surrounded by a sheath.

#### Striatiguttula phoenicis

Taxon classificationFungiPleosporalesStriatiguttulaceae

S.N.Zhang, K.D.Hyde & J.K.Liu, sp. nov.

MB828275

Figure 4

##### Etymology.

The epithet referring to the host on which the fungus was collected.

##### Type.

THAILAND. Ranong: Amphoe Mueang Ranong, Tambon Ngao, on decayed rachis of *Phoenixpaludosa* Roxb. (*Arecaceae*), 6 December 2016, S.N.Zhang, SNT51 (holotype: MFLU 18–1579; isotype: HKAS 97481; ex-type culture MFLUCC 18–0266 = GZCC 18–0008).

##### Description.

*Saprobic* on mangrove date palm *Phoenixpaludosa*. **Sexual morph**: *Ascomata* in vertical section 195–580 µm high, 135–390 µm diameter, (x̄ = 396.0 × 230.3 µm, n = 15), black, scattered, rarely gregarious, immersed, and erumpent through host epidermis by a papilla or a short neck, ampulliform, subglobose, uni-loculate, coriaceous to carbonaceous, ostiolate, periphysate, papillate, glabrous or somewhat interwoven pale brown hyphae or setae, lying around apex of the neck. *Peridium* 10–24 µm thin, composed of several pale brown to hyaline cells of *textura angularis*, compressed and pallid inwardly. Wall of the neck composed thick and elongated angular pale brown to brown cells with hyaline inner layers. *Hamathecium* of 1–2 µm wide, septate, branched, filamentous, anastomosing, trabeculate pseudoparaphyses, embedded in a gelatinous matrix. *Asci* 89–141 × 12–18 µm, (x̄ = 120.5 × 15.4 µm, n = 20), 8-spored, bitunicate, fissitunicate, cylindric-clavate, pedicellate, apically rounded, with an ocular chamber. *Ascospores* 20–29 × 6–10 µm, (x̄ = 24.5 × 7.8 µm, n = 40), hyaline to brown (all cells nearly concolorous), uniseriate to biseriate, fusiform to ellipsoidal, 1–3-septate, constricted at the central septum, the upper middle cell slightly swollen and larger, straight or slightly curved, striate, guttulate, surrounded by an irregular mucilaginous sheath. **Asexual morph**: Undetermined.

**Figure 4.**
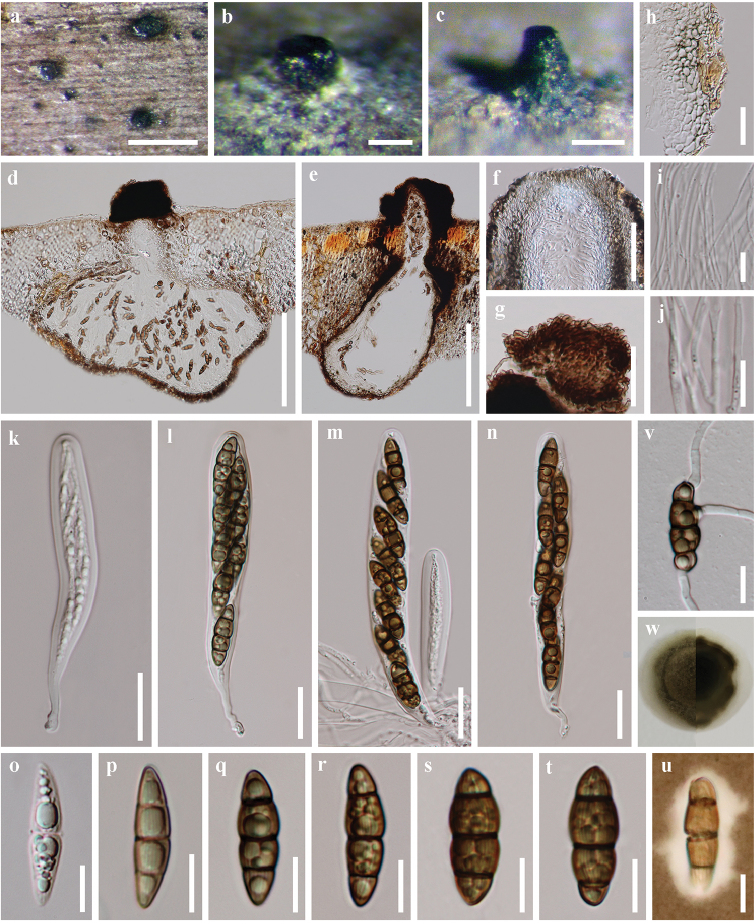
*Striatiguttulaphoenicis* (holotype MFLU 18–1579). **a–c** Appearance of ascoma on host surface **d, e** vertical section through an ascoma **f** ostiole **g** apex of the neck, with somewhat interwoven pale brown hyphae or setae **h** structure of peridium **i, j** pseudoparaphyses **k–n** asci **o–t** ascospores **u** ascospore in India ink and presenting a clear mucilaginous sheath **v** germinating ascospore **w** colony on PDA. Scale bars: 500 μm (**a**), 100 μm (**b, c**), 200 μm (**d, e**), 50 μm **(f, g**), 20 μm (**h, k–n**), 10 μm (**i, j, o–v**).

##### Culture characteristics.

Colonies on PDA attaining 14 mm diam within 21 days at 25 °C under natural light, velvety, centrally raised, greenish grey or greyish olivaceous, reverse dull olivaceous or grey, with a margin of translucent, milky white to hyaline mycelium.

##### Habitat and distribution.

Inhabiting Thai mangrove forests, Andaman sea (west) coastline, Thailand.

##### Notes.

The fusiform to ellipsoidal, 1–3-septate ascospores of *Striatiguttulaphoenicis* is similar to those of *Trematosphaeriamangrovis*, associated with submerged roots of mangrove trees. However, *Striatiguttulaphoenicis* differs from *T.mangrovis* (Kohlmeyer 1968) as the latter has larger ascospores and lacks striations (Table 3). *Striatiguttulaphoenicis* is morphologically different from *S.nypae* as it has ellipsoidal ascospores which are broader in width. Currently, the erumpent to superficial stromata have not been found in *S.phoenicis*. The phylogenetic analysis also confirms that they are distinct species. There are 26 noticeable nucleotide differences across the 474 nucleotides (Suppl. material 1) of ribosomal ITS sequence data (strains: MFLUCC 18–0266 vs. MFLUCC 18–0265, MFLUCC 17–2517 and MFLUCC 17–2518).

#### Longicorpus

Taxon classificationFungiPleosporalesStriatiguttulaceae

S.N.Zhang, K.D.Hyde & J.K.Liu, gen. nov.

MB828276

##### Etymology.

Name refers to the elongated ascomata and ascospores.

##### Description.

*Saprobic* on mangrove palms. **Sexual morph**: *Ascomata* black, scattered to gregarious, immersed, and erumpent through host epidermis by a papilla or a short to long neck, sometimes visible as a slightly raised, dome-shaped area, with a clypeus comprises host tissue and fungal hyphae, ampulliform, subglobose or conical, uni-loculate, coriaceous to carbonaceous, ostiolate, periphysate, papillate, glabrous or somewhat interwoven pale brown hyphae or setae. *Peridium* composing of pale brown or brown angular cells. *Hamathecium* of septate, branched, thin, anastomosing trabeculate pseudoparaphyses, embedded in a gelatinous matrix. *Asci* 8-spored, bitunicate, cylindric-clavate, pedicellate, apically rounded, with an ocular chamber. *Ascospores* uniseriate to biseriate, hyaline to brown, fusiform, 1–3-septate, the upper middle cell slightly swollen towards the central septum, and the end cells paler and smaller, striate, guttulate, surrounded by a mucilaginous sheath. **Asexual morph**: Undetermined.

##### Type species.

*Longicorpusstriataspora* (K.D.Hyde) S.N.Zhang, K.D.Hyde & J.K.Liu.

##### Notes.

**Figure 5.**
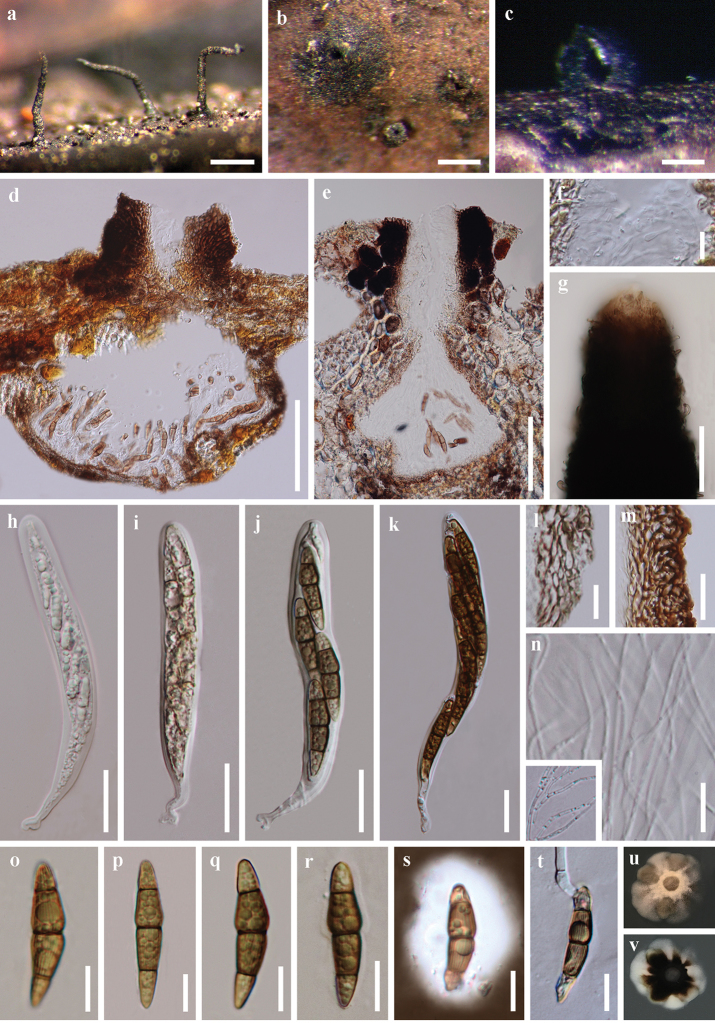
*Longicorpusstriataspora* (epitype MFLU 18–1580, epi-paratype MFLU 18–1582). **a, b** Appearance of ascoma on host surface **c–e** vertical section through an ascoma, with a clypeus near the ostiole **f** ostiole with periphyses **g** apex of the neck, with somewhat interwoven pale brown hyphae or setae **h–k** ascus **l** peridium in vertical section **m** vertical section of the neck, with thicker angular cells **n** pseudoparaphyses **o–r** ascospores **s** ascospore in India ink and presenting a clear mucilaginous sheath **t** germinating ascospore **u, v** Colony on PDA. Scale bars: 500 μm (**a**), 200 μm (**b**), 100 μm (**c–e**), 10 μm (**f, l, n–t**), 50 μm (**g**), 20 μm (**h–k, m**).

*Longicorpus* differs from *Striatiguttula* in having elongate, fusiform ascospores with relatively larger middle cells and paler end cells (Figures 3–5). Multi-gene phylogeny also strongly supports the establishment of two genera. *Longicorpus* is sister to *Striatiguttula* but forms a distinct phylogenetic sub-clade (Figure 1). There are noticeable differences (nucleotide substitutions) at specific positions in the large subunit nuclear ribosomal DNA: 51, 428, 436, 465 (T substituted by C); 53, 55, 102, 153, 163, 166, 251, 367, 369, 427, 435, 440, 446, 448, 466, 504, 550, 654 (C substituted by T); 130 (G substituted by A); 362, 406 (G substituted by T); 370 (C substituted by A); 547 (A substituted by C).

#### Longicorpus striataspora

Taxon classificationFungiPleosporalesStriatiguttulaceae

(K.D.Hyde) S.N.Zhang, K.D.Hyde & J.K.Liu, comb. nov.

MB828277

Figure 5

Trematosphaeria striataspora K.D.Hyde, Botanical Journal of the Linnean Society 98(2): 142. 1988.
Astrosphaeriella striataspora (K.D.Hyde) K.D.Hyde, Botanical Journal of the Linnean Society 110(2): 97. 1992. Type: North Sumatra. K.D.Hyde (holotype: IMI 312390).

##### Epitype.

THAILAND. Ranong: Ranong, on decayed rachis of *Nypafruticans* Wurmb (*Arecaceae*), 6 December 2016, S.N. Zhang, SNT93 (epitype designated here: MFLU 18–1580; epi-isotype designated here: HKAS 97479; ex-epitype living culture MFLUCC 18–0267 = GZCC 18–0009).

##### Description.

*Saprobic* on mangrove palms. **Sexual morph**: *Ascomata* in vertical section (including short papilla) 300–500 µm high, 230–560 µm diameter, (x̄ = 405.3 × 376.6 µm, n = 15), long neck up to 1285 µm, black, scattered to gregarious, immersed, and erumpent through host epidermis by a papilla or a short to long neck, sometimes visible as a slightly raised, dome-shaped area, with a clypeus comprises host tissue and fungal hyphae, ampulliform, subglobose or conical, uni-loculate, coriaceous to carbonaceous, ostiolate, periphysate, papillate, glabrous or somewhat interwoven pale brown hyphae or setae, lying at apex of the neck. *Peridium* 11–15 µm wide, composing of brown to pale brown angular cells, thicker at the rim towards the apex. *Hamathecium* comprising up to 1.5 µm wide, septate, branched, filamentous, trabeculate, anastomosing pseudoparaphyses, embedded in a gelatinous matrix. *Asci* 85–160 × 10–17 µm (x̄ = 122.7 × 13.7 µm, n = 22), 8-spored, bitunicate, cylindric-clavate, pedicellate, apically rounded, with an ocular chamber. *Ascospores* 24–45 × 7–8.8 µm, (x̄ = 34.2 × 7 µm, n = 40), uniseriate to biseriate, hyaline to brown, fusiform, 1–3-septate, the upper middle cell slightly swollen towards the central septate, middle cells larger and longer, end cells paler and smaller, straight or slightly curved, striate, guttulate, surrounded by a mucilaginous sheath. **Asexual morph**: Undetermined.

##### Culture characteristics.

Colonies on PDA attaining 12 mm diameter within 21 days at 25 °C under natural light, velvety, centrally raised, irregular to circular in shape, greenish grey and mixed with milky white mycelium at the edge of a colony, the reverse dull green or grey olivaceous.

##### Additional specimens examined.

Thailand. Chanthaburi, 12°26'43"N, 102°15'47"E, on rachis of *Phoenixpaludosa* Roxb. (*Arecaceae*), immersed mangrove mud and water, 25 April 2017, S.N.Zhang, SNT130 (epi-paratype MFLU 18–1581; living culture MFLUCC 18–0268 = GZCC 18–0010); Thailand. Krabi, near Pali, on decayed rachis of *Nypafruticans* Wurmb (*Arecaceae*), immersed mangrove mud and water, 30 August 2017, S.N.Zhang, SNT195 (epi-paratype MFLU 18–1582; living culture MFLUCC 17–2515 = GZCC 18–0011; MFLUCC 17–2516 = GZCC 18–0012).

##### Habitat and distribution.

Inhabiting in Thai mangrove forests, the Andaman sea (west) coastline and the Gulf of Thailand (east).

##### Notes.

*Longicorpusstriataspora* was found on two mangrove palm species, *Nypafruticans* and *Phoenixpaludosa*. The typical characteristics of *L.striataspora* are the deeply immersed, carbonaceous ascomata with a long neck, and the striate, guttulate, fusiform, 1–3-septate ascospores, with larger middle cells and relatively smaller and paler end cells, surrounded by a mucilaginous sheath. However, such characteristics are similar to *Trematosphaeria* spp. (Table 3), and match with *Trematosphaeriastriataspora* (Hyde 1988), the holotype collected from intertidal wood of *Nypafruticans* in North Sumatra. *Trematosphaeriastriataspora* was later accommodated in *Astrosphaeriella* Syd. & P. Syd. (Hyde 1992a) with proposals for recollection and further phylogenetic studies (Liu et al. 2011b, Phookamsak et al. 2015). We have compared the fresh collections of *Longicorpusstriataspora* with the type material of *Trematosphaeriastriataspora*, and concluded that the two are identical in morphology. On the other hand, the genus *Trematosphaeria* Fuckel has been assigned to the family *Trematosphaeriaceae* K.D. Hyde, Y. Zhang ter, Suetrong & E.B.G. Jones, based on molecular data of its type species *T.pertusa* Fuckel. Therefore, we follow Ariyawansa et al. (2014) and designate an epitype for *Longicorpusstriataspora* in this study.

## Discussion

A novel pleosporalean family, Striatiguttulaceae is introduced herein, which has been compared to several morphologically similar genera and species recovered from mangroves. This study introduces three novel species including an epitypification. The use of divergence times as an additional evidence for ranking taxa (especially in higher taxa ranking) has become possible and several studies have been carried out across different fungal groups (Phukhamsakda et al. 2016, Samarakoon et al. 2016, Divakar et al. 2017, Hongsanan et al. 2017, Hyde et al. 2017, Liu et al. 2017, Zhao et al. 2017). To better understand the placement of Striatiguttulaceae, divergence time was also estimated and this study supports taxonomic schemes proposed earlier. The recent study of ranking a family with divergence time estimates is Liu et al. (2018), who introduced *Lentimurisporaceae*, a new pleosporalean family. We have recovered essentially similar phylogenetic topology, and in an extensive dataset that included berkleasmium-like taxa (referred to Liu et al. 2018), phylogenies generated were also topologically identical to those recovered herein (Figure 1). The monotypic family *Ligninsphaeriaceae* is sister to Striatiguttulaceae, and berkleasmium-like taxa are close to *Aquasubmersaceae*, *Hermatomycetaceae* and *Salsuginaceae* respectively. In this study, the ages of most families in Pleosporales, especially those positioned outside the two suborders were estimated in our divergence time analysis, and the results are comparable to other studies. However, *Ligninsphaeriaceae*, *Pseudoastrosphaeriellaceae* and *Testudinaceae* have relatively younger stem ages than that in Liu et al. (2017), presumably due to different taxa sampling in our phylogeny.

The nature of the pseudoparaphyses (*sensu*Liew et al. 2000) is worth considering here and may provide evidence for separate lineages. The family Striatiguttulaceae, currently with three species, have trabeculate pseudoparaphyses, but also appearing septate. Phylogenetically closely related families of *Ligninsphaeriaceae* and *Pseudoastrosphaeriellaceae* are characterized by cellular pseudoparaphyses and trabeculate pseudoparaphyses respectively.

Considering the ecology of these Striatiguttulaceae species in relation to the mangrove ecosystem, salinity may be an important contributor to their presence. Loilong et al. (2012) have compared fungal community from *Nypafruticans* at different salinities, and found freshwater species in lower salinity and marine species at higher salinity. Although no salinity was measured during our collections, *Longicorpusstriataspora*, *Striatiguttulanypae* and *S.phoenicis* can be considered as manglicolous, because they are found from decayed rachides/petioles of palms, which are perennials submerged in soft mangrove mud and salty water, and well adapted to the varying salinity in mangroves by tidal water. On the other hand, their ascospores have mucilaginous sheaths and lack elaborate appendages, which are also typical characteristics of most mangrove fungi (Jones 2000).

## Supplementary Material

XML Treatment for Striatiguttulaceae

XML Treatment for Striatiguttula

XML Treatment for Striatiguttula nypae

XML Treatment for Striatiguttula phoenicis

XML Treatment for Longicorpus

XML Treatment for Longicorpus striataspora

## Supplementary materials

Supplementary material 1 Phylogenetic analysis This dataset is made available under the Open Database License (http://opendatacommons.org/licenses/odbl/1.0/). The Open Database License (ODbL) is a license agreement intended to allow users to freely share, modify, and use this Dataset while maintaining this same freedom for others, provided that the original source and author(s) are credited.Sheng-Nan Zhang, Kevin D. Hyde, E.B. Gareth Jones, Rajesh Jeewon, Ratchadawan Cheewangkoon, Jian-Kui Liu
