# Supplementary material for: Striatiguttulaceae, a new pleosporalean family to accommodate Longicorpus and Striatiguttula gen. nov. from palms
Source: MycoKeys. 2019 Apr 1;49:99–129. doi: 10.3897/mycokeys.49.30886 (PMC6477835; doi:10.3897/mycokeys.49.30886)
Supplement: Supplementary material 1 [file mycokeys-49-099-s001.doc]

**Supplementary file 1**

>Striatiguttula_phoenicis_MFLUCC_18_0266

TTACCGTTAGGGAACCCACAGGGTCTCCCTGACAAGATAGACCCTTGCCCTTGCATAACC

CCC-GTTTCCTCGGCAGGTCCGCCTGTCGACGGCACAAACAAAACCCCTTCTCCTAAGCA

GCAGTAAA-CGTCACAAAGCACAATTATCAAAACTTTCAACAACGGATCTCTTGGTTCTG

GCATCGATGAAGAACGCAGCGAAATGCGATAAGTAGTGTGAATTGCAGAATTCAGTGAAT

CATCGAATCTTTGAACGCACATTGCGCCCTTTGGTATTCCTTAGGGCATGCCTGTTCGAG

CGTCATTTAACCCCTCAAGCGCGAGCTTGATGTTGGGCGT-TTGTCCCGCCCCAGCGGCG

CGGACTCGCCTCAAAACCATTGGCGGCCCGCGTACCGGCTCAGAGCGCAGCAGATCAGCG

CCTCCGCGCCGGCGCGCCGGCGCCCAGCAAGCCACACATCTTATTT-GACCTCG

>Striatiguttula_nypae_MFLUCC_18_0265

TTACCGTTGGGGGGC-----GAACCCTCCCGACAAGATAGACCCTTGCCCTTGCATAACC

CCCCGTTTCCTCGGCAGGCCCGCCTGCCGACGGCGCAAACCAAAACCCTTCTC-TGAGCA

GCAGTAAAACGTCACAAAGCACAATTATCAAAACTTTCAACAACGGATCTCTTGGTTCTG

GCATCGATGAAGAACGCAGCGAAATGCGATAAGTAGTGTGAATTGCAGAATTCAGTGAAT

CATCGAATCTTTGAACGCACATTGCGCCCTTTGGCATTCCTTAGGGCATGCCTGTTCGAG

CGTCATTCAACCCCTCAAGCGCGAGCTTGATGTTGGGCGTCTTGTCCCGCCCCAGCGGCG

CGGACTCGCCTCAAAGCCATTGGCGGCCCGCGGACCGGCTCAGAGCGCAGCAGATCAGCG

TCTCCGAGCCGGCGCGCCGGCGTCCAGCAAGCCGAAAACCTTATTT-GACCTCG

>Striatiguttula_nypae_MFLUCC_17_2517

TTACCGTTGGGGGGC-----GAACCCTCCCGACAAGATAGACCCTTGCCCTTGCATAACC

CCCCGTTTCCTCGGCAGGCCCGCCTGCCGACGGCGCAAACCAAAACCCTTCTC-TGAGCA

GCAGTAAAACGTCACAAAGCACAATTATCAAAACTTTCAACAACGGATCTCTTGGTTCTG

GCATCGATGAAGAACGCAGCGAAATGCGATAAGTAGTGTGAATTGCAGAATTCAGTGAAT

CATCGAATCTTTGAACGCACATTGCGCCCTTTGGCATTCCTTAGGGCATGCCTGTTCGAG

CGTCATTCAACCCCTCAAGCGCGAGCTTGATGTTGGGCGTCTTGTCCCGCCCCAGCGGCG

CGGACTCGCCTCAAAGCCATTGGCGGCCCGCGGACCGGCTCAGAGCGCAGCAGATCAGCG

TCTCCGAGCCGGCGCGCCGGCGTCCAGCAAGCCGAAAACCTTATTTTGACCTCG

>Striatiguttula_nypae_MFLUCC_17_2518

TTACCGTTGGGGGGC-----GAACCCTCCCGACAAGATAGACCCTTGCCCTTGCATAACC

CCCCGTTTCCTCGGCAGGCCCGCCTGCCGACGGCGCAAACCAAAACCCTTCTC-TGAGCA

GCAGTAAAACGTCACAAAGCACAATTATCAAAACTTTCAACAACGGATCTCTTGGTTCTG

GCATCGATGAAGAACGCAGCGAAATGCGATAAGTAGTGTGAATTGCAGAATTCAGTGAAT

CATCGAATCTTTGAACGCACATTGCGCCCTTTGGCATTCCTTAGGGCATGCCTGTTCGAG

CGTCATTCAACCCCTCAAGCGCGAGCTTGATGTTGGGCGTCTTGTCCCGCCCCAGCGGCG

CGGACTCGCCTCAAAGTCATTGGCGGCCCGCGGACCGGCTCAGAGCGCAGCAGATCAGCG

TCTCCGAGCCGGCGCGCCGGCGTCCAGCAAGCCGAAAACCTTATTTTGACCTCG
